# Single versus dual antibiotic-loaded bone cement in hip fracture arthroplasty: a cohort study of 56,434 hip arthroplasties from the Dutch Arthroplasty Register

**DOI:** 10.2340/17453674.2026.46527

**Published:** 2026-08-13

**Authors:** Seung-Jae YOON, Laura VAN MARLE, Liza N VAN STEENBERGEN, Pieter K BOS, Paul C JUTTE, Marjan WOUTHUYZEN-BAKKER, Wierd P ZIJLSTRA

**Affiliations:** 1 Department of Orthopaedic Surgery, University Medical Center Groningen, University of Groningen, Groningen; 2 Department of Orthopaedic Surgery, Frisius Medical Center, Leeuwarden; 3 Dutch Arthroplasty Register, Landelijke Registratie Orthopedische Interventies (LROI), ‘s Hertogenbosch; 4 Department of Orthopedics and Sports Medicine, Erasmus Medical Center, Rotterdam; 5 Department of Medical Microbiology and Infection Prevention, University Medical Center Groningen, University of Groningen, Groningen, the Netherlands

## Abstract

**Background and purpose:**

Hip fracture treatment with (hemi)arthroplasty carries a risk of periprosthetic joint infection (PJI), which may be reduced by using dual antibiotic-loaded bone cement (ALBC). We aimed to compare the rate of all-cause revision, revision for infection, and mortality following single or dual ALBC use in hip fracture arthroplasty.

**Methods:**

From the Dutch Arthroplasty Register, we identified all cemented hemiarthroplasties (HA) and total hip arthroplasties (THA) performed in the period 2007–2024 for proximal femoral fracture. Crude cumulative incidences of all-cause and infection revision were estimated using competing risk survival analyses with death as competing event. Mortality was estimated using Kaplan–Meier analysis. Cause-specific Cox regression models adjusting for age, year of surgery, history of previous surgeries, and American Society of Anesthesiologists (ASA) classification estimated hazard ratios (HRs).

**Results:**

We analyzed 56,434 hip arthroplasties, of which 1,651 used dual ALBC. 1-year crude cumulative incidences were similar across groups for all-cause revision, infection revision, and mortality. After adjustment, dual ALBC was not associated with a reduced rate of all-cause revision (HR 1.2, 95% confidence interval [CI] 0.9–1.5]) compared with single ALBC. Dual ALBC was associated with a modestly higher revision for infection (HR 1.6, CI 1.0–2.5) and mortality in HA (HR 1.2, CI 1.1–1.2).

**Conclusion:**

Dual ALBC was not associated with lower revision rates compared with single ALBC but a modestly higher revision rate was noted for infection and mortality following hip arthroplasty after hip fracture.

Hip fractures are among the most common injuries in older adults, placing a substantial burden on patients and healthcare systems [1]. Guidelines recommend treating displaced femoral neck fractures with hemiarthroplasty (HA) or total hip arthroplasty (THA), depending on factors such as patient activity level and life expectancy [2–6]. Periprosthetic joint infection (PJI) is a serious complication that hinders recovery, being associated with 1-year mortality rates of up to 40% [7]. Notably, the incidence of PJI in fracture arthroplasties is 3–4%, twice the rate observed in elective arthroplasties for osteoarthritis [8]. The impact and frequency of PJI in this vulnerable population highlight the need for effective preventive strategies.

One proposed surgical strategy is the use of dual antibiotic-loaded bone cement (ALBC), which typically combines high doses of gentamicin with clindamycin or vancomycin. This approach is thought to enhance antibiotic elution and broaden antimicrobial coverage compared with single low-dose ALBC, which commonly contains gentamicin. However, despite its theoretical advantages, clinical evidence remains inconclusive regarding whether dual ALBC provides superior protection against PJI compared with single ALBC [9-12]. Moreover, dual ALBC is associated with greater cost and may potentially induce antibiotic resistance [13-15].

Therefore, we aimed to compare the rate of all-cause revision, revision for infection, and mortality following single or dual ALBC use in hip fracture arthroplasty.

## Methods

### Study design and data source

This study is reported according to the STROBE guidelines. This observational study used data from the Dutch Arthroplasty Register (LROI), a nationwide population-based registry that has recorded primary and revision arthroplasties since 2007 with high completeness (96% for primary HA, 98% for primary THA, and 99% for revision hip arthroplasties) [16]. For each procedure, data on patient, procedure, and implant characteristics were available.

### Participants

All cemented hip arthroplasties performed for fracture and registered in the LROI between 2007 and 2024 were assessed for eligibility. Arthroplasties for acute hip fracture and as reoperation for complications after osteosynthesis were included. Procedures were excluded if they used non-antibiotic (plain) bone cement. Cases with missing information on cement type or on variables required for the Cox regression models were also excluded.

### Outcomes

Revision was defined as the replacement, addition, or removal of 1 or more prosthetic components. The reason for revision is recorded for all revision procedures in the LROI. Revision for infection was defined by a preoperative diagnosis or suspicion of PJI, as recorded by the surgeon. To ascertain mortality, the registry links its records to the national healthcare insurance database Vektis, which provides up-to-date information on patients’ survival status and date of death.

### Statistics

Descriptive statistics of baseline patient characteristics were reported by procedure type and cement group. Categorical variables were compared using the chi-square test, and continuous variables using the Mann–Whitney U test.

Competing risk survival analyses were used to estimate the cumulative incidence for all-cause revision and revision for infection, with death treated as a competing risk. Survival time was calculated from the date of primary surgery to revision surgery, death, or administrative censoring on January 1, 2025. The incidence of mortality was calculated using Kaplan–Meier survival analysis. For each outcome (all-cause revision, revision for infection, and mortality), 1-, 2-, and 5-year cumulative incidence was reported. Associations between the use of dual ALBC and each outcome were assessed using multivariable Cox proportional hazards regression models. Covariates included in the models were age, year of surgery, history of previous surgeries, and American Society of Anesthesiologists (ASA) classification. The proportional hazards assumption was evaluated graphically using log-log survival plots. Results are presented as hazard ratios (HRs) with 95% confidence intervals (CIs).

Several sensitivity analyses were performed to assess the robustness of the findings. First, all analyses were repeated after stratifying by procedure type (hemiarthroplasty and total hip arthroplasty), to account for possible differences in treatment effects between procedures. Second, multivariable Cox proportional hazards models were refitted with additional adjustment for body mass index (BMI) and smoking status. As these variables were only registered from 2014, this analysis was conducted in a reduced cohort from the period 2014 to 2023 with complete data for these covariates. Third, survival analyses were repeated in a propensity score-matched cohort. Propensity scores for receiving dual ALBC were estimated using logistic regression based on the covariates included in the primary multivariable models. Patients were matched 1:1 using nearest-neighbor matching with a caliper width of 0.2 of the standard deviation of the logit of the propensity score, without replacement. Cox proportional hazards regression was then performed in the matched cohort to estimate hazard ratios for each outcome.

For all tests, a 2-tailed significance level of P < 0.05 was used. Statistical analyses were performed using IBM SPSS Statistics version 28 (IBM Corp, Armonk, NY, USA) and R version 4.4.3 (R Foundation for Statistical Computing, Vienna, Austria).

### Ethics, registration, use of AI tools, funding, and disclosures

Approval for the use of registry data in this study was provided by the LROI Scientific Advisory Committee and LROI board. Under the Dutch Medical Research Involving Human Subjects Act (WMO), formal ethical review was not required as all data was fully anonymized. This study received no external funding. Artificial intelligence tools were used only for writing assistance in the preparation of this manuscript. SY and MWB received a research grant from Heraeus Medical GmbH for work unrelated to the present study. PKB received lecture honoraria from the AO Foundation, Stryker, and Radboud University. Complete disclosure of interest forms according to ICMJE are available on the article page, doi: 10.2340/17453674.2026.46527

## Results

**Figure 1:**
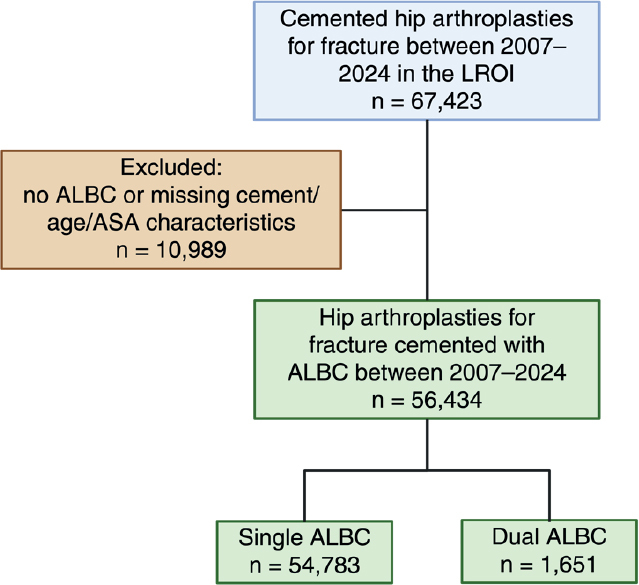
Flowchart of hemiarthroplasty and total hip arthroplasty procedures for hip fracture in the Dutch Arthroplasty Register (LROI) database, 2007–2024. ALBC: antibiotic-loaded bone cement. ASA: American Society of Anesthesiologists. Created in BioRender. Yoon, C. (2026) https://BioRender.com/skjvyl1

After exclusion based on the use of non-antibiotic (plain) bone cement (n = 191) or missing data (n = 10,798), 56,434 hip arthroplasties registered between 2007 and 2024 were included for analysis (Figure 1). Dual ALBC was used in 2.9% of procedures, while the remaining 97% of procedures used single ALBC. Dual ALBC was used significantly more often in recent years, with 71% of dual ALBC cases performed since 2018 compared with 61% of single ALBC cases.

### Baseline characteristics

Minor differences in baseline characteristics were observed between patients receiving dual and single ALBC, with standardized mean differences (SMD) below 0.15 (Table 1). The dual ALBC group had a slightly higher proportion of smokers (11% vs 7.7%, SMD = 0.15), hemiarthroplasties (88% vs 83%, SMD = 0.15), and previous surgeries (3.1% vs 1.8%, SMD = 0.12) than the single ALBC group. The groups did not show clinically relevant differences in age (81 vs 82, SMD = 0.11), BMI (median 24 vs 24, SMD = 0.07), or ASA classification (SMD = 0.03). Nearly all single ALBC formulations contained gentamicin (98%). Among dual ALBC formulations, the most used antibiotics were gentamicin and clindamycin (59%).

**Table 1:** Characteristics of patients receiving hip arthroplasty for fracture. Values are count (%) unless otherwise specified

Variable	Total cohort	Single ALBC	Dual ALBC	SMD
No. of procedures	56,434	54,783	1,651	–
Median age (IQR)	82 (75–87)	82 (75–87)	81 (73–86)	0.11
Male sex	18,773 (33)	18,183 (33)	590 (36)	0.05
Median BMI (IQR)	24.2 (22.0–26.7)	24.2 (22.0–26.7)	23.8 (21.3–26.8)	0.07
Smoker	4,376 (7.8)	4,203 (7.7)	173 (10)	0.15
Type of operation				0.15
Hemiarthroplasty	46,646 (83)	45,199 (82)	1,447 (88)	
Total hip arthroplasty	9,788 (17)	9,584 (18)	204 (12)	
Previous surgeries	1,044 (1.8)	983 (1.8)	61 (3.1)	0.12
ASA classification				0.03
I	1,875 (3.3)	1,827 (3.3)	48 (2.9)	
II	19,973 (35)	19,402 (35)	571 (35)	
III–IV	34,586 (61)	33,554 (61)	1,032 (62)	
Median FU, years (IQR)	2.4 (0.8–5.0)	2.4 (0.8–4.9)	2.0 (0.6–4.2)	0.13
Antibiotics in bone cement				–
Gentamicin	53,601 (95)	53,601 (98)	0 (0)	
Tobramycin	1,182 (2.1)	1,182 (2.2)	0 (0)	
Gentamicin + clindamycin	979 (1.7)	0 (0)	979 (59)	
Erythromycin + colistin	563 (1.0)	0 (0)	563 (34)	
Gentamicin + vancomycin	99 (0.2)	0 (0)	99 (6.0)	
Gentamicin + tobramycin	7 (0.0)	0 (0)	7 (0.4)	

ALBC: antibiotic-loaded bone cement, BMI: body mass index, IQR: interquartile range,

FU: follow-up, SMD: standardized mean difference.

### All-cause revision

**Figure 2:**
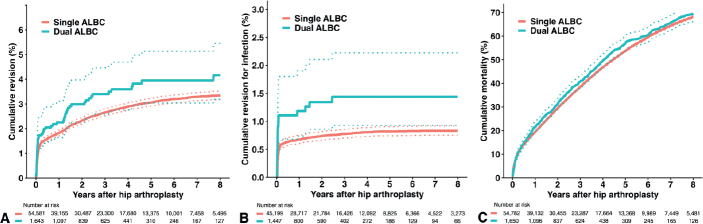
Crude cumulative incidence of (A) all-cause revision, (B) revision for infection, and (C) mortality after hip arthroplasty for fracture, stratified by the type of antibiotic-loaded bone cement (ALBC). Created in BioRender. Yoon, C. (2026) https://BioRender.com/ksfwh0k

Cumulative incidence curves did not show significantly different all-cause revision rates between single and dual ALBC recipients (Figure 2). The 1-year incidence was 1.8% (CI 1.7–1.9) for single ALBC compared with 2.3% (CI 1.6–3.1) for dual ALBC, while the 5-year incidence was 3.1% (CI 2.9–3.2) and 3.9 (CI 3.0–5.1), respectively (Table 2). Adjusted analyses demonstrated a non-significant positive association between dual ALBC and the rate of revision (HR 1.2, CI 0.9–1.5) (Table 3).

**Table 2:** Crude cumulative incidence (%) with 95% confidence interval of all-cause revision, revision for infection, and mortality 1, 2, and 5 years after hip arthroplasty for fracture, stratified by type of antibiotic-loaded bone cement (ALBC)

Factor	Single ALBC (n = 54,783)	Dual ALBC (n = 1,651)
All-cause revision		
1 year	1.8 (1.7–1.9)	2.3 (1.6–3.1)
2 years	2.3 (2.2–2.5)	3.0 (2.2–4.0)
5 years	3.1 (2.9–3.2)	3.9 (3.0–5.1)
Revision for infection		
1 year	0.7 (0.6–0.7)	1.2 (0.7–1.9)
2 years	0.7 (0.7–0.8)	1.3 (0.9–2.1)
5 years	0.8 (0.7–0.9)	1.4 (0.9–2.2)
Mortality		
1 year	19.5 (19.1–19.8)	21.7 (19.6–23.7)
2 years	29.5 (29.1–29.9)	31.2 (28.8–33.6)
5 years	53.8 (53.3–54.3)	56.8 (53.8–59.8)

**Table 3:** Adjusted hazard ratios with 95% confidence interval (CI) of all-cause revision, revision for infection, and mortality by type of antibiotic-loaded bone cement (ALBC). Cause-specific Cox regression adjusted for age, procedure year, previous surgeries, and ASA classification

Double vs single ALBC	Adjusted HR (CI)
All-cause revision	1.2 (0.9–1.5)
Revision for infection	1.6 (1.0–2.5)
Mortality	1.2 (1.1–1.2)

### Revision for infection

1-year cumulative incidence of revision for infection was 0.7% (CI 0.6–0.7) for single ALBC and 1.2% (CI 0.7–1.9) for dual ALBC. At 5 years, cumulative incidences were 0.8% (CI 0.7–0.9) and 1.4% (CI 0.9–2.2), respectively. Dual ALBC was associated with an increased rate of revision for infection after adjustment (HR 1.6, CI 1.0–2.5).

### Mortality

For mortality, the cumulative incidences with single ALBC and dual ALBC were 19.5% (CI 19.1–19.8) and 21.7% (CI 19.6–23.7) at 1 year, and 53.8% (CI 53.3–54.3) and 56.8% (CI 53.8–59.8) at 5 years, respectively. In the adjusted model, dual ALBC use was associated with an increased mortality rate (HR 1.2, CI 1.1–1.2).

### Sensitivity analyses

Results were consistent across all sensitivity analyses. Analyses on hemiarthroplasties and total hip arthroplasties separately showed similar estimates to the primary analysis (Supplementary data). Additional adjustment for BMI and smoking status in multivariable Cox models did not substantially change the direction or magnitude of the associations between dual ALBC use and all outcomes in either hemiarthroplasty or total hip arthroplasty. Likewise, analyses performed in the propensity score-matched cohorts yielded hazard ratio estimates comparable to those of the primary analyses, with no statistically significant associations between dual ALBC and all-cause revision or revision for infection, and similar findings for mortality.

## Discussion

We aimed to compare the rate of all-cause revision, revision for infection, and mortality between single and dual ALBC use in hip fracture arthroplasty in the first nationwide analysis in the Netherlands. After adjustment for measured confounders, dual ALBC was not associated with a reduced rate of all-cause revision. It was associated with a modest increase in revision for infection and mortality, and these findings were consistent across sensitivity analyses.

Several observational studies and a quasi-randomized trial, mostly from the United Kingdom, have reported a lower risk of deep surgical site infection with dual ALBC, and pooled estimates suggest a protective effect [10,11,17,18]. A German registry study by Szymski et al. [12] reported slightly but not significantly lower 5-year infection rates with dual ALBC (1.5%, CI 0.7–2.4) than with single ALBC (2.3%, CI 2.1–2.6). However, the most robust evidence to date comes from the large UK-based WHiTE 8 trial [9] of 4,936 older adults aged ≥ 60 years, which found no statistically significant difference (adjusted odds ratio for single vs dual ALBC, 1.43, CI 0.87–2.35; P = 0.2). A subsequent Bayesian reanalysis reported a 92% posterior probability that the relative risk exceeded 1 and an 81% posterior probability of an absolute risk difference of at least 0.25%, both favoring dual ALBC [19]. A Nordic study will soon present its results on the topic [20]. Together, these figures suggest a probable but modest benefit.

Our results may be compatible with the emerging literature. However, interpretation requires careful consideration of indication bias. The Dutch Arthroplasty Register does not capture the clinical rationale for selecting single or dual ALBC, and the factors driving cement choice therefore remain unknown. Measured baseline characteristics were broadly comparable between groups, suggesting against substantial confounding by recorded factors. However, the antibiotic content of cement has no plausible effect on mortality; the elevated mortality associated with dual ALBC is therefore most likely explained by residual confounding, presumably reflecting greater frailty or unmeasured comorbidity among patients selected for dual ALBC. Given this, the hazard ratio for infection revision (HR 1.6, CI 1.0–2.5) is notable. It showed a higher rate associated with dual ALBC, despite a hypothesized protective effect. Given that residual confounding likely biases these estimates away from showing benefit, a modest protective effect, as seen in the literature, cannot be excluded. However, a large reduction in revision for infection is more difficult to reconcile with this data, even after accounting for indication bias.

From a clinical perspective, the absence of an increase in all-cause revision is reassuring in settings where dual ALBC is already routine, and the higher infection-related estimate is more plausibly attributable to confounding than to a true effect of the cement. Our data nonetheless does not demonstrate sufficient benefit to recommend adoption where single ALBC is used. Clinicians should also weigh the higher cost of dual ALBC and the possibility of antibiotic resistance associated with widespread local antibiotic use [13-15]. A cost-utility analysis performed on the WhiTE 8 trial data concluded that dual ALBC was unlikely to be more cost-effective than single ALBC in fracture hemiarthroplasty, with a negative net monetary benefit [13]. Cement selection should thus continue to be guided by local protocols and judgment until further evidence emerges, such as from the ongoing register-based cluster-randomized DAICY trial [20].

### Limitations

First, although we adjusted for available confounders including BMI and smoking status, other factors such as nutritional status, surgeon and hospital preferences for cement, and other preventive measures such as staphylococcal decolonization protocols were not recorded [21]. Second, as previously stated, the registry does not capture indications for cement selection, which restricts our ability to fully adjust for confounding by indication. Third, the LROI only captures revisions involving component exchange, meaning infections managed with suppressive antibiotics or debridement without component exchange are not recorded. Of the revisions that are captured, only those with a preoperative diagnosis or suspicion of infection are registered by the surgeon as revision for infection. As a result, the LROI captures approximately one-third of all PJIs [22], and our revision and infection figures likely underestimate the true incidence [23]. Fourth, dual ALBC was more frequently used in recent years, resulting in a shorter follow-up for this group. Fifth, we did not formally adjust for multiple comparisons. We regarded the hazard ratios for all-cause revision, revision for infection, and mortality as our primary endpoints, with the stratified and sensitivity analyses considered exploratory. As such, isolated significant findings should be interpreted with caution [24]. Lastly, there were imbalances in the type of procedure across groups, with hemiarthroplasties comprising a higher proportion in the dual ALBC group. Nonetheless, our sensitivity analyses stratified by procedure type showed similar effect estimates in hemiarthroplasties and total hip arthroplasties.

### Conclusion

Dual ALBC was not associated with lower revision rates compared with single ALBC but a modestly higher revision rate was noted for infection and mortality following hip arthroplasty after hip fracture. Put in perspective, even though dual ALBC was relatively rare and potentially reserved for higher-risk patients in this registry, these findings do not support a routine shift toward dual ALBC in this setting.

### Supplementary data

Tables S1–S2 and Figures S1–S3 are available as supplementary data on the article page, doi: 10.2340/17453674.2026.46527

## Supplementary Material



## Notes

SY: conceptualization, statistical analysis, writing the original draft, reviewing the manuscript. LvM: statistical analysis, reviewing the manuscript. LNvS: data collection, reviewing the manuscript. PKB: conceptualization, reviewing the manuscript. PCJ: reviewing the manuscript, supervision. MWB: conceptualization, reviewing the manuscript, supervision. WPZ: conceptualization, writing the original draft, reviewing the manuscript, supervision.

The authors would like to thank the Dutch Arthroplasty Register (LROI) for providing the data used in this study.

Handling co-editors: Marianne Westberg and Robin Christensen

*Acta* thanks Håvard Dale and Anna Stefánsdóttir for help with peer review of this manuscript.
