# Supplementary material for: Single versus dual antibiotic-loaded bone cement in hip fracture arthroplasty: a cohort study of 56,434 hip arthroplasties from the Dutch Arthroplasty Register
Source: Acta Orthop. 2026 Aug 13;97:538–43. doi: 10.2340/17453674.2026.46527 (PMC13469801; doi:10.2340/17453674.2026.46527)
Supplement: Supplementary file 1 [file ActaO-97-46527-s1.pdf]

Supplementary Data

Table S1. Crude cumulative incidence (%) and 95% confidence interval of all-cause revision, revision for infection, and mortality 1, 2, and 5 years after hemiarthroplasty or total hip arthroplasty for hip fracture, stratified by type of antibiotic-loaded bone cement (ALBC).

		Hemiarthroplasty		Total hip arthroplasty	
		**Single ALBC (n = 45,199)**	**Dual ALBC (n = 1,447)**	**Single ALBC (n = 9,584)**	**Dual ALBC (n = 204)**
**All-cause revision**	1 year	1.7 (1.6–1.8)	2.2 (1.5–3.1)	2.4 (2.1–2.7)	2.9 (1.3–6.4)
	2 years	2.2 (2.1–2.3)	2.7 (1.9–3.7)	2.9 (2.6–3.3)	5.0 (2.7–9.1)
	5 years	2.8 (2.7–3.0)	3.7 (2.7–4.9)	4.2 (3.8–4.6)	6.0 (3.5–10.4)
**Revision for infection**	1 year	0.6 (0.5–0.7)	1.1 (0.6–1.8)	0.9 (0.8–1.2)	2.0 (0.7–5.2)
	2 years	0.7 (0.6–0.7)	1.2 (0.7–1.9)	1.1 (0.9–1.4)	2.5 (1.1–5.9)
	5 years	0.7 (0.6–0.8)	1.3 (0.8–2.1)	1.4 (1.2–1.7)	2.5 (1.1–5.9)
**Mortality**	1 year	22.5 (22.1–22.9)	24.2 (21.9–26.5)	4.8 (4.3–5.2)	5.0 (2.0–8.1)
	2 years	33.9 (33.5–34.4)	34.4 (31.8–37.1)	8.0 (7.5–8.6)	10.3 (6.0–14.6)
	5 years	60.5 (60.0–61.0)	63.0 (59.7–66.3)	19.9 (18.9–20.9)	24.3 (17.9–30.8)

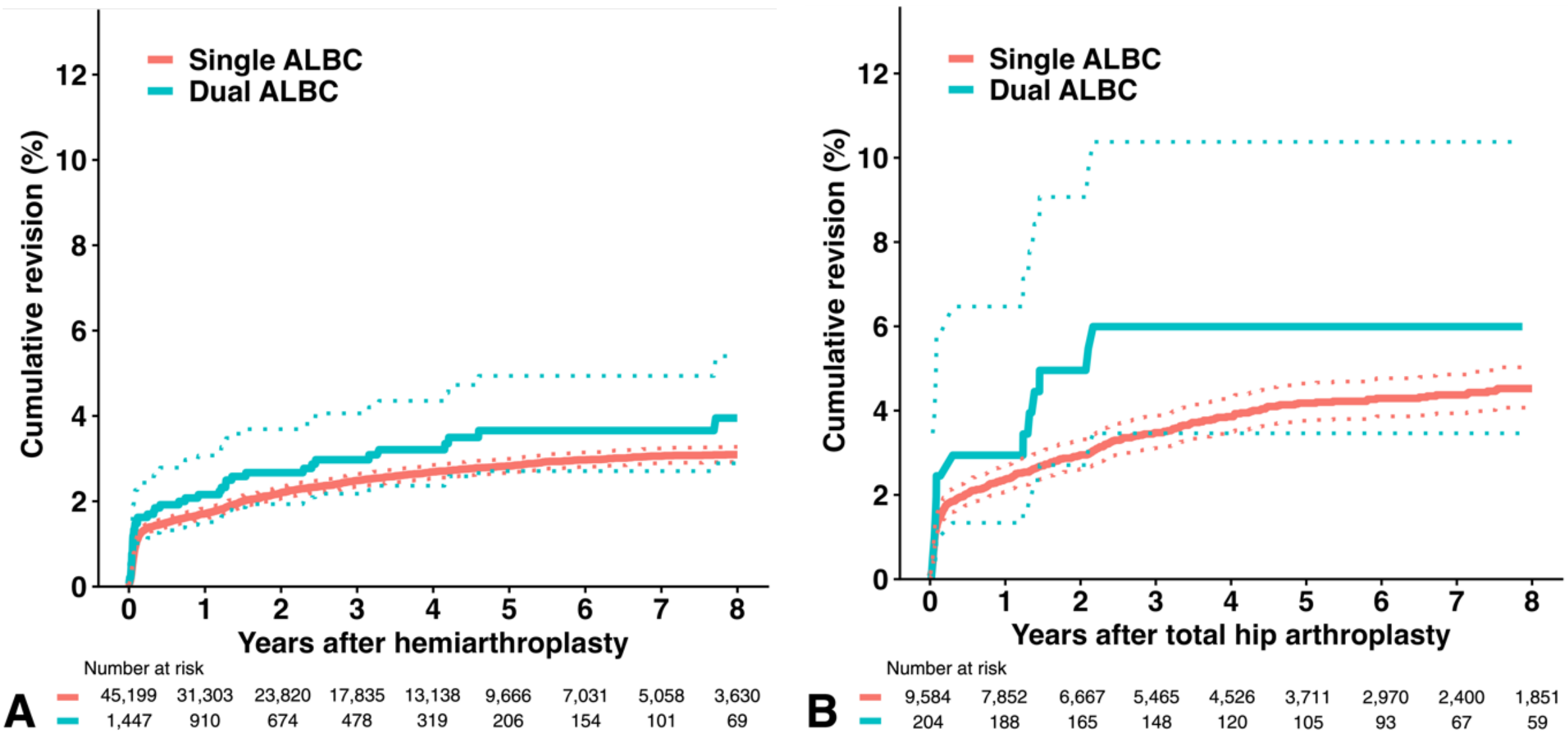

Figure S1. Cumulative incidence of all-cause revision after (A) hemiarthroplasty and (B) total hip arthroplasty for fracture, stratified by the type of antibiotic-loaded bone cement (ALBC).

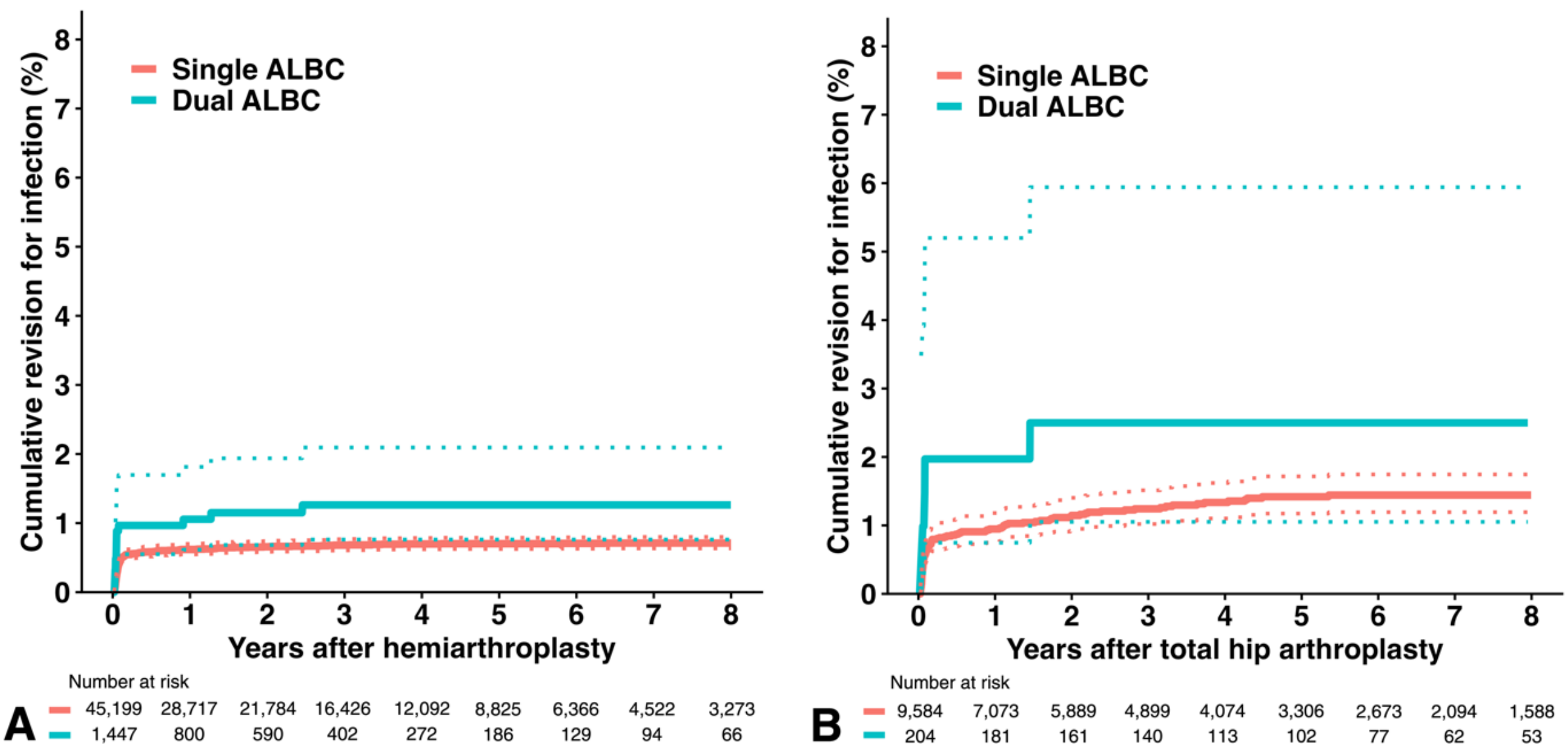

Figure S2. Cumulative incidence of revision for infection after (A) hemiarthroplasty and (B) total hip arthroplasty for fracture, stratified by the type of antibiotic-loaded bone cement (ALBC).

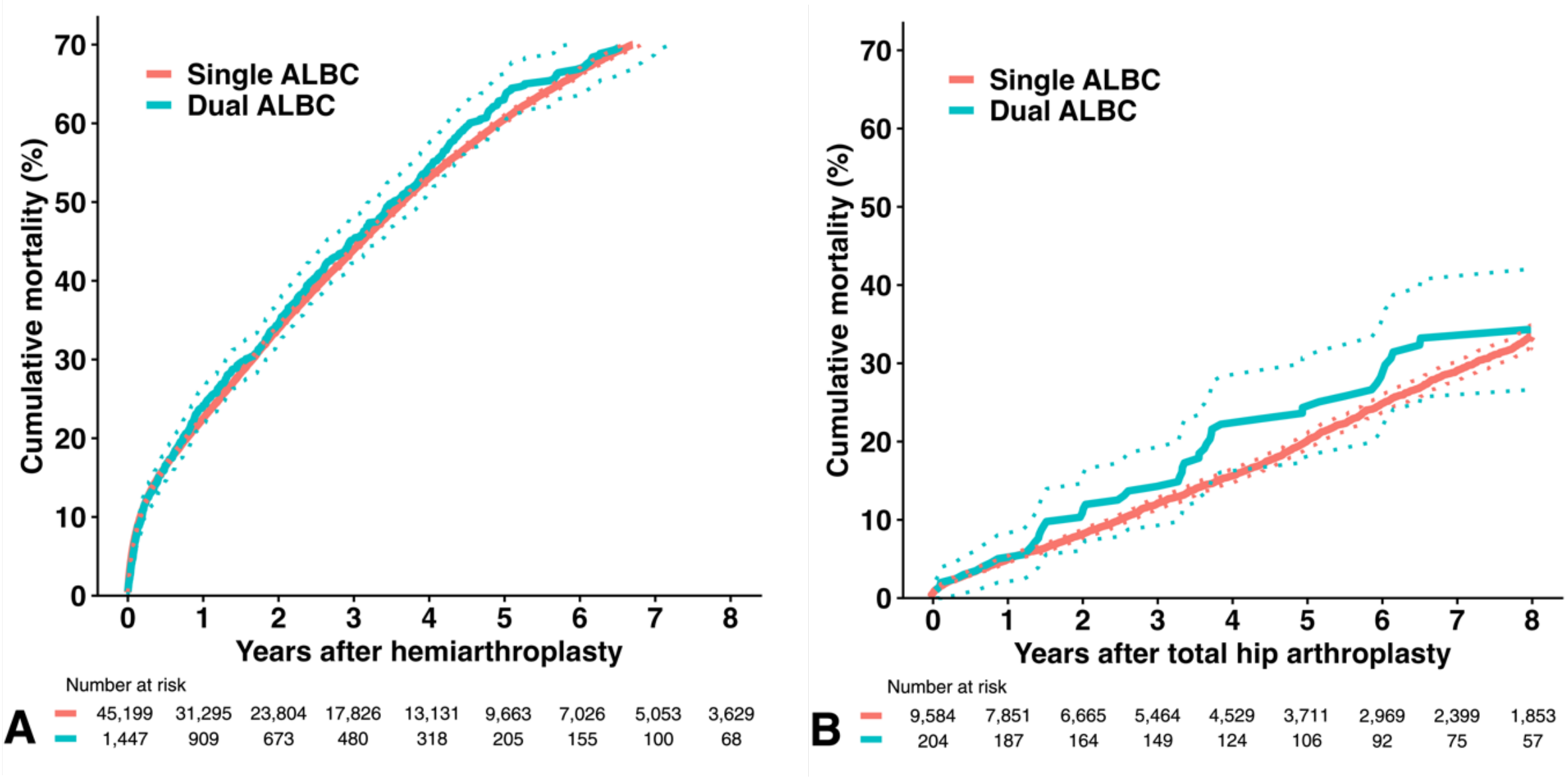

Figure S3. Cumulative incidence of mortality after (A) hemiarthroplasty and (B) total hip arthroplasty for fracture, stratified by the type of antibiotic-loaded bone cement (ALBC).

Table S2. Adjusted hazard ratios and 95% confidence interval of all-cause revision, revision for infection, and mortality by type of antibiotic-loaded bone cement (ALBC). Cause-specific Cox regression adjusted for age, procedure year, previous surgeries, and ASA classification

	Hemiarthroplasty		Total hip arthroplasty	
**Adjusted HR**	**Dual ALBC**	**P value**	**Dual ALBC**	**P value**
All-cause revision	1.1 (0.8–1.5)	0.5	1.1 (0.6–2.2)	0.7
Revision for infection	1.5 (0.9–2.6)	0.1	1.8 (0.7–4.4)	0.2
Mortality	1.1 (1.0–1.2)	<0.01	1.2 (0.9–1.6)	0.3
